# The long-term prognostic implications of free triiodothyronine to free thyroxine ratio in patients with obstructive sleep apnea and acute coronary syndrome

**DOI:** 10.3389/fendo.2024.1451645

**Published:** 2024-09-16

**Authors:** Yun Zhou, Qian He, Hui Ai, Xuedong Zhao, Xiuhuan Chen, Siyi Li, Zekun Zhang, Jingyao Fan, Wei Gong, Shaoping Nie

**Affiliations:** ^1^ Center for Coronary Artery Disease, Division of Cardiology, Beijing Anzhen Hospital, Capital Medical University, Beijing, China; ^2^ National Clinical Research Center for Cardiovascular Diseases, Beijing, China; ^3^ Beijing Institute of Heart, Lung, and Blood Vessel Diseases, Beijing, China; ^4^ Department of Cardiology, Beijing Hospital, National Center of Gerontology, Institute of Geriatric Medicine, Chinese Academy of Medical Sciences, Beijing, China

**Keywords:** acute coronary syndrome, obstructive sleep apnea, FT3/FT4 ratio, thyroid hormones, prognosis

## Abstract

**Objective:**

Obstructive sleep apnea (OSA) and thyroid dysfunction frequently overlap clinically and are risk factors for cardiovascular disease. The free triiodothyronine to free thyroxine (FT3/FT4) ratio as a novel biomarker of cardiovascular disease prognosis, but the impact of the FT3/FT4 ratio on the prognosis of OSA in patients with acute coronary syndromes (ACS) remains uncertain.

**Methods:**

In this prospective cohort study, 2160 patients with ACS were recruited and underwent portable sleep monitoring at Beijing Anzhen Hospital from June 2015 to January 2020. OSA was diagnosed when apnea-hypopnea index of ≥15 events/h. Patients were further divided into tertiles according to FT3/FT4 ratio. All patients had scheduled follow-up visits at 1, 3, 6, 9 and 12 months after discharge, with subsequent outpatient visits or telephone follow-up visits every 6 months. The primary endpoint was major adverse cardiovascular and cerebrovascular events (MACCE), including cardiovascular death, myocardial infarction (MI), stroke, ischemia-driven revascularization, or hospitalization for unstable angina or heart failure.

**Results:**

Among 1,547 euthyroid patients enrolled (mean age, 56.0 ± 10.5 years), 812 patients (52.5%) had OSA. The FT3/FT4 ratio between OSA and non-OSA patients was not significantly different. During 2.8 (1.4, 3.5) years follow up, the risk of MACCE increased with the decreasing FT3/FT4 tertiles in patients with OSA (tertile3 as reference, tertile2: hazard ratio (HR) 1.26, 95% CI: 0.85-1.86, P = 0.255; tertile1: 1.60, 95% CI 1.11-2.32; P = 0.013). After adjustment for confounders, the lowest FT3/FT4 tertile was still independently associated with an increased risk of MACCE (adjusted HR 1.66, 95% CI 1.11-2.50, P = 0.015).

**Conclusion:**

Lower FT3/FT4 ratio associated with poor prognosis in patients with ACS and OSA.

## Introduction

Obstructive sleep apnea (OSA) is a common condition where repetitive breathing pauses expose the cardiovascular system to cycles of hypoxia, excessive negative intrathoracic pressure, and arousal. OSA affected approximately 34% and 17% man and women in middle age respectively. OSA can promote the progression of atherosclerosis and is strongly associated with poor prognosis in patients with acute coronary syndromes (ACS) (1, 2). In previous studies, 50% ~ 65% of patients with ACS suffered from OSA, and the presence of OSA also predicted adverse cardiovascular events following ACS in different subgroups (3–5), but the mechanism remains unclear.

OSA and thyroid dysfunction frequently overlap clinically, and the effect of abnormal thyroid hormones (TH) levels on the cardiovascular system is well-established. Previous studies have indicated that overt and subclinical thyroid dysfunction (6, 7) as well as low triiodothyronine (T3) syndrome (8) can increase the risk of coronary artery disease (CAD). Due to the widespread presence of thyroid hormone receptors in myocardial tissue, even subtle fluctuations within the reference range of thyroid hormone levels can lead to the deterioration of specific clinical conditions (9). This could potentially be attributed to impaired peripheral TH deiodination and downregulation of deiodinase activity (10, 11). A lower free triiodothyronine to free thyroxine (FT3/FT4) ratio reflects decreased tissue-specific deiodinase activity and has been demonstrated to be a novel biomarker for predicting the prognosis of several cardiovascular diseases (12–14).

However, the impact of OSA on TH in patients with ACS is unclear, and the effects of TH alterations on clinical prognosis remain uncertain. In this study, we aimed to investigate the association of the FT3/FT4 ratio with long-term cardiovascular events in patients with ACS with or without OSA.

## Methods

### Study design and participants

This is a sub-study of the OSA-ACS project (www.clinicaltrials.gov.identifier.NCT03362385), which is a prospective, observational, single-center study to evaluate the association between OSA and cardiovascular outcomes in patients with ACS. Consecutive eligible patients aged 18 to 85 years were admitted for ACS with an overnight sleep study at Beijing Anzhen Hospital, Capital Medical University, spanning from June 2015 to January 2020. ACS was defined as the acute presentation of coronary disease, including ST-segment elevation myocardial infarction (STEMI), non-ST-segment elevation myocardial infarction (NSTEMI), and unstable angina. The exclusion criteria included: (1) cardiac arrest or cardiogenic shock; (2) history of malignancy; (3) failed sleep monitoring (inadequate or unsatisfactory signal recording); (4) patients with predominantly central sleep apnea (≥ 50% central events and central apnea-hypopnea index (AHI) of ≥ 10 breaths/hour) and those already undergoing regular continuous positive airway pressure treatment (> 4 hours/day or > 21 days/month); (5) patients without detailed data of thyroid function tests; (6) patients with prior/current thyroid disorders.

This study adheres to the recommendations of the Helsinki Declaration and obtained approval from the Ethics Committee of Beijing Anzhen Hospital, Capital Medical University (No. 2013025). Written informed consent was provided by all participants.

### Sleep study and management

All registered participants underwent an overnight sleep study utilizing a type III portable cardiorespiratory polygraphy device (ApneaLink Air; ResMed) following clinical stabilization during hospitalization. Devices were applied by trained researchers before bedtime, and data was collected the next morning. Outputs were recorded by researchers unaware of clinical characteristics in an OSA database. Sleep studies adhered to American Academy of Sleep Medicine standards (15). Recorded signals included nasal airflow, chest and abdominal movements, snoring episodes, heart rate, and oxygen saturation (SaO_2_). Apnea was defined as an absence of airflow for ≥ 10 seconds. Hypopnea was defined as a 30% reduction in airflow for ≥ 10 seconds along with a > 4% decrease in arterial SaO_2_. AHI was defined as the number of apneas and hypopneas recorded per hour. Following current guidelines and recommendations (16, 17), OSA was defined as AHI ≥ 15 events·h^−1^. Patients with AHI < 15 events·h^−1^ were considered as the non-OSA group. Within each group, further stratification was performed based on the tertile distribution of the FT3/FT4 ratio. The Hypoxemic burden was quantified by calculating oxygen desaturation index (ODI) per hour of sleep and the percentage of time with SaO_2_ < 90% (18). We used the Epworth Sleepiness Scale to analyze patients' self-reported levels of daytime sleepiness.

According to current guidelines (17, 18), all patients received standard treatment during their hospitalization for ACS. Percutaneous Coronary Intervention (PCI) with stent implantation or coronary artery bypass grafting (CABG) is performed when surgery is deemed necessary. Patients with AHI ≥15, particularly those with excessive daytime sleepiness, were referred to a sleep center for further evaluation.

### Determination of serum thyroid function

All participants had fasting blood samples drawn within 24 hours of admission. Thyroid function was assessed using a direct chemiluminescent method (ADVIA Centaur, Siemens, USA). Reference ranges were as follows: FT3, 3.28-6.47 pmol/L; FT4, 7.64-16.03 pmol/L; thyroid-stimulating hormone (TSH), 0.49-4.91 mIU/L; total T3, 1.01-2.48 nmol/L; total thyroxine (T4), 67.97-152.52 nmol/L; If TSH, FT3, and FT4 fall within the normal reference range, thyroid function is considered normal.

### Endpoints and follow-up

All patients underwent follow-up at 1, 3, 6, 9, and 12 months post-discharge, followed by subsequent outpatient visits or telephone follow-ups every 6 months. Follow-up and the adjudication of all clinical events were conducted by an independent Clinical Events Committee blinded to the patient’s clinical characteristics and sleep results.

The primary endpoint was major adverse cardiovascular and cerebrovascular events (MACCE), defined as a composite of cardiovascular death, recurrent myocardial infarction (MI), stroke, ischemia-driven revascularization, or composite events requiring hospitalization due to unstable angina or heart failure. The secondary endpoints were the components of MACCE and all-cause death. All endpoints were defined by the definitions proposed by the Cardiovascular Trials Standardized Data Collection Initiative (19) and have been previously described (3, 4). All events were independently assessed by adjudicators unaware of the results of the sleep study. Adjudicators also reviewed source documents and determined the necessity of hospitalization and/or revascularization.

### Statistical analysis

Quantitative data were shown as mean ± standard deviation or median (first, third quartiles) and assessed using Student’s t-test or Mann-Whitney U test. Qualitative data were presented as percentages (%) and assessed using χ^2^ statistics or Fisher exact test. Stratification based on the FT3/FT4 ratio was performed to generate Kaplan-Meier curves for the OSA and non-OSA groups and compared by log-rank test. The Cox proportional hazards regression model was employed to determine whether FT3/FT4 ratio was an independent predictor of events, stratified by the OSA or OSA-related characteristics status. Confounding factors with potential clinically relevant endpoints or that showed a univariate relationship with endpoints were adjusted in multivariable models, including age, sex, body mass index (BMI), smoking, hypertension, diabetes, dyslipidemia, clinical presentation, low-density lipoprotein cholesterol (LDL-C), and TSH. Assessment of nonlinear associations of FT3/FT4 ratio with MACCE using the restricted cubic spline (RCS) method (3 nodes, the median as a reference point).

The results were presented as hazard ratio (HR) and 95% confidence intervals (95% CI). A two-sided P-value < 0.05 was considered significant. All statistical analyses were performed using SPSS 25 (IBM, Armonk, NY, USA) and R version 4.3.2((R Institute for Statistical Computing, Vienna, Austria).

## Results

### Demographic and clinical characteristics

**Figure 1:**
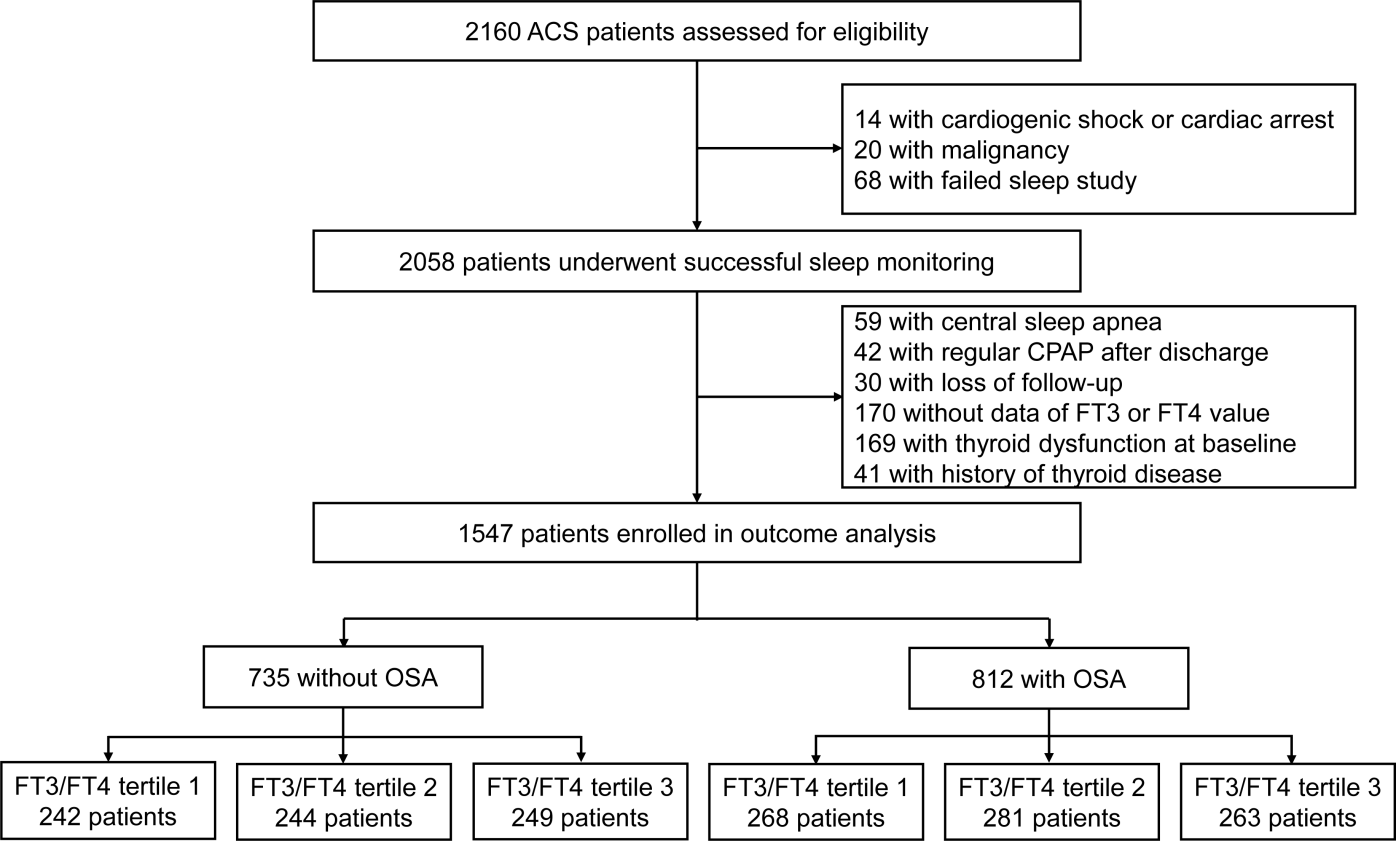
Study flowchart. CPAP, continues positive airway pressure; FT3, free triiodothyronine; FT4, free thyroxine; OSA, obstructive sleep apnea.

A total of 1,547 patients with ACS were included in the final analysis and 812 patients (52.5%) had OSA. The TH and FT3/FT4 ratio between OSA and non-OSA patients were not significantly different (all P > 0.05, **Supplementary Table 1**). Further stratification was performed based on the tertile distribution of the FT3/FT4 ratio (Tertile 1 < 0.4, 0.4 ≤ Tertile 2 < 0.48, Tertile 3 ≥ 0.48). The study flow chart is shown in **Figure 1**. Baseline demographic and clinical characteristics according to FT3/FT4 ratio tertiles are presented in **Supplementary Table 2**. Patients in the lowest tertile of FT3/FT4 ratio were significantly older and less likely to be male than in the other two groups (all P < 0.05). Furthermore, the reduction of FT3/FT4 ratio was associated with lower BMI, diastolic blood pressure, estimated glomerular filtration rate (eGFR), left ventricular ejection fraction (LVEF), but higher high sensitivity to C-reactive proteins (Hs-CRP), brain natriuretic peptide (BNP) and prevalence of prior stroke, and was more likely to be diagnosed with STEMI (all P <0.05). However, the prevalence of OSA and sleep monitoring indicators were not significantly different in the FT3/FT4 ratio tertiles (**Supplementary Table 3**).

Baseline demographic and clinical characteristics by FT3/FT4 ratio tertiles and OSA categories are shown in **Table 1**. Irrespective of OSA status, patients with the lowest tertile of FT3/FT4 ratio remained older, had lower BMI, neck circumference and triglycerides, and had more clinical diagnoses of STEMI. In the OSA group, compared with the middle and highest tertile of the FT3/FT4 ratio, patients in the lowest tertile of the FT3/FT4 ratio were more likely to be female, to have a history of stroke, to be current smokers, and to have lower blood pressure, LVEF, eGFR, total cholesterol, low-density lipoprotein, but higher BNP, hemoglobin A1C and Hs-CRP. In the non-OSA group, patients in the lowest tertile of FT3/FT4 ratio were more likely to have a lower waist-to-hip ratio but a higher prevalence of prior CABG than the other two groups. In both the OSA and non-OSA groups, other characteristics were generally well-matched.

**Table 1:** Demographic and clinical characteristics by FT3/FT4 ratio tertiles and OSA categories.

Variables	Non-OSA (n=735)			*P* value	OSA (n=812)			*P* value
	Tertile 1 (n=242)	Tertile 2 (n=244)	Tertile 3 (n=249)		Tertile 1 (n=268)	Tertile 2 (n=281)	Tertile 3 (n=263)	
Subjects								
Age, years	58.7 ± 9.8	55.0 ± 9.9	54.2 ± 10.2	<0.001	59.5 ± 10.7	55.1 ± 11.0	53.7 ± 9.8	<0.001
Male	191(78.9)	209(85.7)	214(85.9)	0.061	226(84.3)	247(87.9)	247(93.9)	0.002
BMI, kg/m^2^	25.2 ± 3.4	26.1 ± 3.3	26.6 ± 3.1	<0.001	27.1 ± 3.3	28.2 ± 3.5	28.8 ± 3.7	<0.001
Waist-to-hip ratio	0.96(0.93-1)	0.98(0.94-1)	0.98(0.94-1.01)	0.04	0.99(0.96-1.02)	0.99(0.96-1.02)	0.99(0.96-1.03)	0.382
Neck circumference, cm	39(37-41)	40(38-42)	40(38-42)	<0.001	41(38-43)	42(39-44)	42(40-45)	<0.001
Systolic BP, mmHg	125(117-138)	127(120-140)	126(118-136)	0.497	125(115-137)	127(117-140)	130(120-140)	0.008
Diastolic BP, mmHg	74(69-82)	75(70-83)	76(70-84)	0.068	75(70-84)	76(70-85)	80(72-89)	<0.001
Medical history								
Diabetes	110(45.5)	99(40.6)	111(44.6)	0.511	138(51.5)	130(46.3)	108(41.1)	0.055
Hypertension	166(68.6)	164(67.2)	156(62.7)	0.345	197(73.5)	208(74)	195(74.1)	0.984
Hyperlipidaemia	79(32.6)	82(33.6)	87(34.9)	0.864	78(29.1)	105(37.4)	94(35.7)	0.099
Prior stroke	28(11.6)	27(11.1)	20(8)	0.373	49(18.3)	27(9.6)	21(8)	<0.001
Prior MI	33(13.6)	43(17.6)	34(13.7)	0.363	51(19)	51(18.1)	44(16.7)	0.785
Prior PCI	44(18.2)	49(20.1)	40(16.1)	0.511	70(26.1)	57(20.3)	61(23.2)	0.269
Prior CABG	6(2.5)	0(0)	2(0.8)	0.021	4(1.5)	5(1.8)	4(1.5)	0.957
Current Smoking	107(44.2)	121(49.6)	125(50.2)	0.608	113(42.2)	148(52.7)	140(53.2)	0.006
Baseline tests								
eGFR, mL·min-1·1.73 m^-2^	105(87.9-123)	106.7(90-126.1)	108.4(93.7-124.4)	0.213	99.7(82.4-116.3)	103.9(90.8-120.2)	107.3(90.5-122.1)	0.001
HbA1c, %	6(5.6-7.1)	5.9(5.6-6.7)	6(5.6-7)	0.614	6.2(5.7-7.8)	6(5.6-7.1)	6.1(5.6-6.9)	0.043
hsCRP,mg/L	1.4(0.63-5.74)	1.8(0.68-4)	1.23(0.52-3.31)	0.057	3.61(1.2-11.34)	2.31(0.94-6.38)	2.2(1.04-6)	0.002
BNP, pg/mL	50.0(19.9-172.8)	41.0(19.9-93.5)	31.9(19.9-70.9)	<0.001	73.0(28.0-189.0)	48.0(19.9-127.4)	32.0(19.0-75.9)	0.064
LVEF, %	62(55-66)	62(57-65)	63(59-66)	0.129	60(55-65)	61(58-65)	62(58-66)	0.003
TC, mmol/L	4.02(3.31-4.82)	4.06(3.46-4.93)	4.2(3.41-4.98)	0.252	3.99(3.46-4.79)	4.17(3.55-5.01)	4.29(3.72-5.04)	0.003
TG, mmol/L	1.35(0.94-1.9)	1.58(1.15-2.23)	1.52(1.15-2.33)	<0.001	1.39(1.06-1.94)	1.61(1.17-2.4)	1.83(1.3-2.65)	<0.001
LDL-C, mmol/L	2.34(1.73-3.01)	2.38(1.86-3.11)	2.4(1.82-3.11)	0.469	2.37(1.89-3.03)	2.48(1.94-3.13)	2.58(2.06-3.15)	0.036
HDL-C, mmol/L	1.01(0.85-1.19)	1.01(0.84-1.16)	1.03(0.89-1.18)	0.333	0.98(0.85-1.14)	0.96(0.85-1.13)	0.98(0.86-1.13)	0.605
TSH, mIU/L	1.49(1.06-2.29)	1.74(1.14-2.43)	1.67(1.14-2.36)	0.169	1.74(1.12-2.48)	1.59(1.15-2.38)	1.73(1.13-2.48)	0.601
FT3, pmol/L	4.44(4.12-4.78)	4.83(4.52-5.14)	5.17(4.80-5.57)	<0.001	4.41(4.06-4.79)	4.85(4.51-5.2)	5.21(4.88-5.63)	<0.001
FT4, pmol/L	12.47(11.34-13.56)	11.2(10.37-11.9)	9.80(8.94-10.49)	<0.001	12.47(11.56-13.54)	11.13(10.45-12.02)	9.90(9.09-10.55)	<0.001
**Diagnosis**				0.032				<0.001
STEMI	56(23.1)	41(16.8)	39(15.7)		94(35.1)	63(22.4)	46(17.5)	
NSTEMI	47(19.4)	55(22.5)	38(15.3)		44(16.4)	59(21)	45(17.1)	
Unstable angina	139(57.4)	148(60.7)	172(69.1)		130(48.5)	159(56.6)	172(65.4)	
Procedures								
PCI	146(60.3)	104(42.6)	145(58.2)	0.793	170(63.4)	189(67.3)	176(66.9)	0.583
CABG	16(6.6)	24(9.8)	21(8.4)	0.434	244(91)	13(4.6)	249(94.7)	0.083
Medications on discharge								
Aspirin	233(96.3)	238(97.5)	244(98)	0.484	258(96.3)	275(97.9)	258(98.1)	0.348
P2Y_12_ inhibitors	223(92.1)	216(88.5)	224(90)	0.4	246(91.8)	262(93.2)	243(92.4)	0.811
β-Blockers	175(72.3)	185(75.8)	186(74.7)	0.665	219(81.7)	224(79.7)	202(76.8)	0.372
ACEIs/ARBs	144(59.5)	141(57.8)	137(55)	0.598	179(66.8)	187(66.5)	170(64.6)	0.848
Statins	238(98.3)	238(97.5)	247(99.2)	0.349	266(99.3)	276(98.2)	255(97)	0.145

The data is presented as mean ± standard deviation, median (first quartile to third quartile), or n (%). ACEI, angiotensin-converting enzymes inhibitor; ARB, angiotensin receptor blocker; BMI, body mass index; BP, blood pressure; CABG, coronary artery bypass grafting; FT3, free triiodothyronine; FT4, free thyroxine; Hs-CRP, High sensitivity C-reactive protein; HDL-C, high-density lipoprotein cholesterol; LDL-C, low-density lipoprotein cholesterol; LVEF, left ventricular ejection fraction; NSTEMI, Non-ST-segment-elevation myocardial infarction; PCI, percutaneous coronary intervention; STEMI, ST-segment-elevation myocardial infarction; TSH, thyroid stimulating hormone; UA, unstable angina.

When the effect of the FT3/FT4 ratio tertiles on sleep monitoring indicators was further compared stratified by the presence or absence of OSA, among patients without OSA, a reduction in the FT3/FT4 ratio was associated with a higher mean SaO_2_ and a lower Epworth Sleep Scale (ESS). The differences in the other indicators were not insignificant statistically (**Table 2**).

**Table 2:** Overnight sleep monitoring results stratified by the interaction term between OSA and FT3/FT4 ratio tertiles.

Variables	Non-OSA (n=735)			*P* value	OSA (n=812)			*P* value
	Tertile 1 (n=242)	Tertile 2 (n=244)	Tertile 3 (n=249)		Tertile 1 (n=268)	Tertile 2 (n=281)	Tertile 3 (n=263)	
Sleep study								
AHI, events·h^−1^	8(4-11)	7(4-11)	8(5-11)	0.143	28(20.3-40)	29(22-43)	29(20-43)	0.315
ODI, events·h^−1^	8.6(5.0-11.4)	8(4.1-11.6)	8.7(5.3-12)	0.166	27.2(19.3-36.5)	27.5(21.1-39.5)	28(19.4-41.9)	0.414
Minimum SaO_2_, %	88(85-90)	88(85-90)	87(84-89)	0.095	83(77-86)	83(78-86)	82(77-86)	0.245
Mean SaO_2_, %	95(93-96)	94.85(94-95)	94(93-95)	0.001	93(92-94)	93(92-94)	93(92-94)	0.922
T90, %	0.5(0-3)	0.4(0.1-1.85)	1(0.1-3)	0.065	5.3(2-15)	7(2-14)	7(2-18)	0.537
Epworth Sleepiness Scale	6(2-9)	7(3-11)	7(3-11)	0.041	9(5-12)	8(4-12)	8(5-12)	0.552

The data is presented as median (first quartile to third quartile); AHI, apnea-hypopnea index; ODI, oxygen desaturation index; SaO_2_, arterial oxygen saturation; T90=percentage of Time with SaO_2_ <90%.

### Outcomes in the overall population according to OSA status and FT3/FT4 tertiles

The median follow-up time was 2.8 years (1.4, 3.5), during which 306 cases of MACCE occurred (**Supplementary Table 4**). In patients with ACS, the presence of OSA was associated with a higher incidence of MACCE compared to individuals without OSA (Log-rank, P =0.003; **Supplementary Figure 1**). Patients with lower levels of the FT3/FT4 ratio (lowest tertile) had significantly higher rates of cardiovascular death (HR:3.37, 95% CI: 1.12-10.17, P =0.031) and hospitalization for heart failure (HR: 10.90, 95% CI: 1.43-83.19, P =0.021) compared to those with higher levels (highest tertile). The incidence of MACCE was also elevated in patients with lower levels of the FT3/FT4 ratio, but the difference was not statistically significant (HR: 1.31, 95% CI: 0.99-1.74, P = 0.060) (**Supplementary Table 4**, **Supplementary Figure 2**).

### Outcomes of patients in FT3/FT4 tertiles stratified by OSA

**Figure 2:**
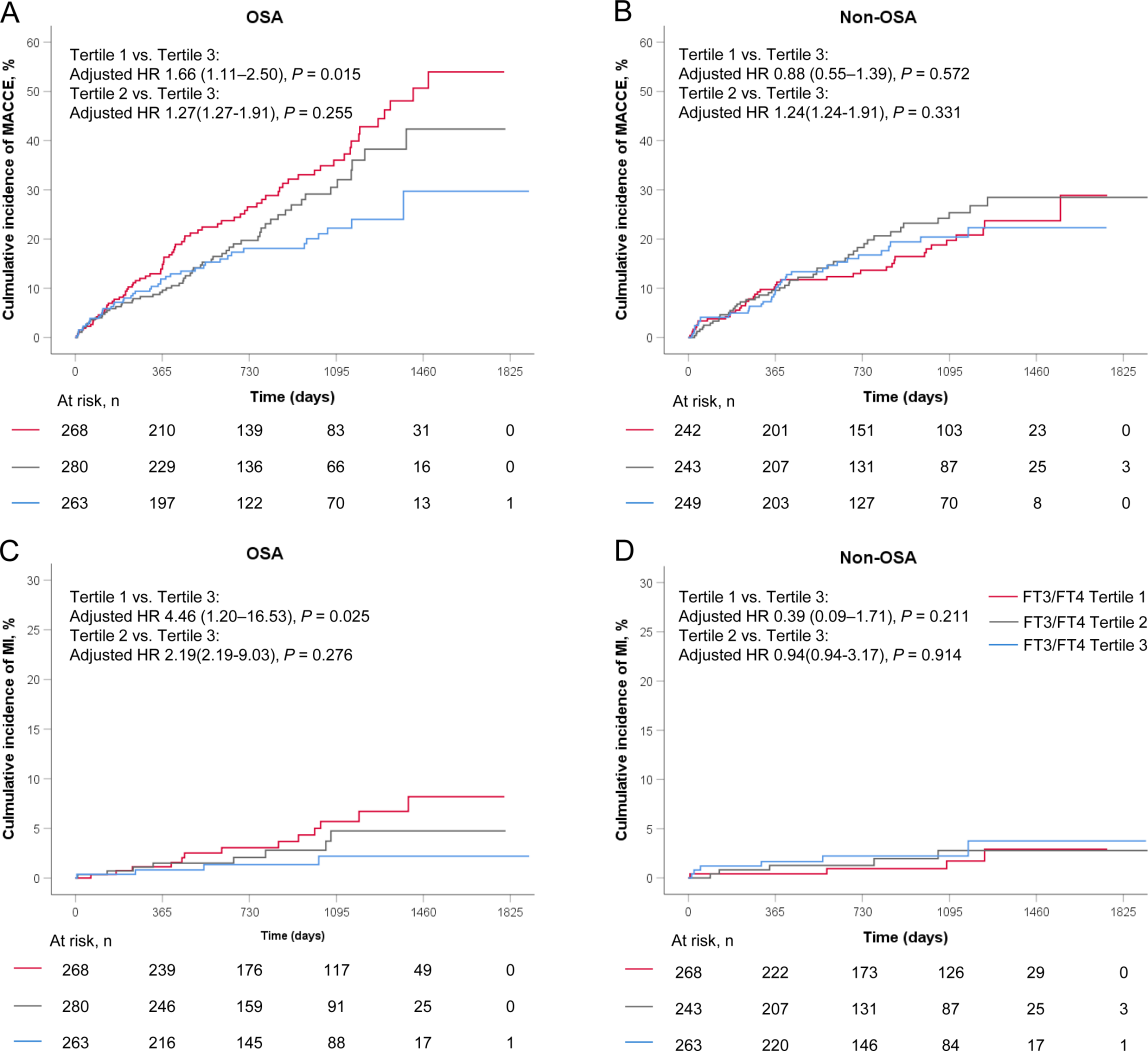
Kaplan-Meier curves in FT3/FT4 tertiles stratified by OSA for **(A, B)** MACCE, and **(C, D)** MI. MACCE, major adverse cardiovascular and cerebrovascular event; MI, myocardial infarction; OSA, obstructive sleep apnea.

In the OSA group, the incidence of MACCE rises from the highest to the lowest tertile of FT3/FT4 ratio (tertile 3 as reference; tertile 2: 1.26, 95% CI: 0.85-1.86, P = 0.255; tertile 1: HR 1.60, 95%CI 1.11-2.32; P = 0.013; **Table 3**), but in the non-OSA group, there were no differences in the incidence of MACCE among the three groups(tertile 3 as reference; tertile 2: HR 1.15, 95%CI 0.75-1.76, P = 0.522; tertile 1:HR 0.98; 95%CI 0.63-1.52; P = 0.931; **Table 3**). After adjustment for age, sex, BMI, smoking, hypertension, diabetes, dyslipidemia, clinical presentation, LDL-C, and TSH, the results revealed that a lower FT3/FT4 ratio was associated with an increased risk of MACCE in the OSA group (tertile 3 as reference; tertile 2: adjusted HR 1.27, 95% CI 0.84-1.91, P = 0.255; tertile 1: adjusted HR 1.66, 95% CI 1.11-2.50, P = 0.015; **Figure 2A**, **Table 3**), but not in the non-OSA group (**Figure 2B**, **Table 3**). Recurrent MI was significantly elevated from the highest to the lowest tertile of the FT3/FT4 ratio (tertile 3 as reference; tertile 2: adjusted HR 2.19, 95% CI 0.53-9.03, P = 0.276; tertile 1: adjusted HR 4.46, 95% CI 1.20-16.53, P = 0.025; **Figure 2C**, **Table 3**) in the patients with OSA. However, in the non-OSA group, there was no difference in the incidence of MI among the three groups (**Figure 2D**, **Table 3**). No significant interaction was noted between the tertiles of FT3/FT4 ratio and OSA with respect to MACCE (interaction P = 0.070). Other secondary endpoints showed no significant difference among the tertiles of FT3/FT4 ratio in patients with or without OSA. There was no significant interaction between the tertiles of FT3/FT4 ratio and OSA for those secondary end-points (interaction P ≥ 0.070 for all).

**Table 3:** Cox regression analysis for clinical outcomes in FT3/FT4 ratio tertiles stratified by OSA.

Clinical outcomes	Group		Events (n, %)	Unadjusted		Adjusted^*^	
				HR (95% CI)	*P* value	HR (95% CI)	*P* value
**MACCE**	OSA	Tertile 1	77(28.7)	1.60(1.11-2.32)	0.013	1.66(1.11-2.50)	0.015
		Tertile 2	26(23.6)	1.26(0.85-1.86)	0.255	1.27(0.84-1.91)	0.255
		Tertile 3	44(16.7)	1(ref)		1(ref)	
	NON-OSA	Tertile 1	41(16.9)	0.98(0.63-1.52)	0.931	0.88(0.55-1.39)	0.572
		Tertile 2	17(16.3)	1.15(0.75-1.76)	0.522	1.24(0.80-1.91)	0.331
		Tertile 3	39(15.7)	1(ref)		1(ref)	
**Recurrent MI**	OSA	Tertile 1	13(4.9)	2.70(0.88-8.33)	0.083	4.46(1.20-16.53)	0.025
		Tertile 2	6(5.5)	1.88(0.57-6.24)	0.303	2.19(0.53-9.03)	0.276
		Tertile 3	4(1.5)	1(ref)		1(ref)	
	NON-OSA	Tertile 1	4(1.7)	0.58(0.16-2.08)	0.406	0.39(0.09-1.71)	0.211
		Tertile 2	1(1.0)	0.81(0.25-2.64)	0.721	0.94(0.28-3.17)	0.914
		Tertile 3	6(2.4)	1(ref)		1(ref)	
**Cardiovascular death**	OSA	Tertile 1	8(3.0)	3.20(0.68-15.14)	0.142	3.18(0.56-17.92)	0.19
		Tertile 2	4(3.6)	2.79(0.56-13.81)	0.209	3.49(0.65-18.68)	0.144
		Tertile 3	2(0.8)	1(ref)		1(ref)	
	NON-OSA	Tertile 1	7(2.9)	3.57(0.74-17.2)	0.112	3.04(0.61-15.24)	0.177
		Tertile 2	3(2.9)	2(0.37-10.93)	0.423	2.18(0.35-13.52)	0.402
		Tertile 3	2(0.8)	1(ref)		1(ref)	
**Hospitalization for UA**	OSA	Tertile 1	44(16.4)	1.29(0.81-2.05)	0.281	1.12(0.67-1.86)	0.66
		Tertile 2	16(14.5)	1.28(0.8-2.03)	0.302	1.22(0.75-1.97)	0.421
		Tertile 3	31(11.8)	1(ref)		1(ref)	
	NON-OSA	Tertile 1	22(9.1)	0.68(0.39-1.18)	0.17	0.68(0.38-1.21)	0.19
		Tertile 2	12(11.5)	1.08(0.66-1.76)	0.773	1.15(0.70-1.91)	0.581
		Tertile 3	30(12.0)	1(ref)		1(ref)	
**Stroke**	OSA	Tertile 1	11(4.1)	1.36(0.53-3.53)	0.524	1.50(0.53-4.22)	0.443
		Tertile 2	1(0.9)	0.66(0.21-2.09)	0.483	0.55(0.17-1.83)	0.333
		Tertile 3	7(2.7)	1(ref)		1(ref)	
	NON-OSA	Tertile 1	5(2.1)	1.60(0.38-6.71)	0.52	1.64(0.36-7.49)	0.525
		Tertile 2	3(2.9)	2.68(0.71-10.12)	0.145	3.63(0.87-15.1)	0.076
		Tertile 3	3(1.2)	1(ref)		1(ref)	
**Ischemia-driven revascularization**	OSA	Tertile 1	32(11.9)	1.61(0.90-2.87)	0.108	1.60(0.85-3.04)	0.149
		Tertile 2	10(9.1)	1.12(0.6-2.09)	0.721	0.99(0.51-1.93)	0.99
		Tertile 3	18(6.8)	1(ref)		1(ref)	
	NON-OSA	Tertile 1	14(5.8)	0.64(0.32-1.27)	0.2	0.54(0.26-1.11)	0.094
		Tertile 2	5(4.8)	0.87(0.46-1.64)	0.664	0.95(0.50-1.81)	0.871
		Tertile 3	20(8.0)	–	–	–	–
**Hospitalization for HF**	OSA	Tertile 1^+^	7(2.6)	–	–	–	–
		Tertile 2^+^	1(0.9)	–	–	–	–
		Tertile 3^+^	1(0.4)	–	–	–	–
	NON-OSA	Tertile 1^+^	7(2.9)	–	–	–	–
		Tertile 2^+^	0(0)	–	–	–	–
		Tertile 3^+^	0(0.0)	–	–	–	–
**All-cause death**	OSA	Tertile 1	8(3.0)	2.15(0.57-8.15)	0.26	1.47(0.32-6.81)	0.621
		Tertile 2	5(4.5)	2.16(0.56-8.35)	0.264	2.44(0.59-10.15)	0.22
		Tertile 3	3(1.1)	1(ref)		1(ref)	
	NON-OSA	Tertile 1	8(3.3)	1.60(0.52-4.9)	0.408	1.42(0.40-5.02)	0.586
		Tertile 2	6(5.8)	1.6(0.52-4.89)	0.409	2.35(0.67-8.19)	0.181
		Tertile 3	5(2.0)	1(ref)		1(ref)	

CI, confidence interval; HR, hazard ratio; MACCE, major adverse cardiovascular and cerebrovascular event; MI, myocardial infarction; OSA, obstructive sleep apnea; UA, unstable angina. ^*^: adjusted for age (<65, ≥65), sex (male or female), BMI (<28, ≥28), smoking (no, past, current), hypertension (yes or no), diabetes (yes or no), dyslipidemia (yes or no), clinical presentation (STEMI, NSTE-ACS); LDL-C (continuous); TSH (continuous). ^+^: univariate and/or multivariate Cox regression was not done due to no or few number of events.

The RCS model revealed similar results, with a monotonically increasing risk of MACCE with decreasing levels of the FT3/FT4 ratio in all patients and in patients with OSA, but not in patients without OSA (**Supplementary Figure 3**). The inflection point of the RCS curve was determined to be at FT3/FT4 ratio = 0.5. Based on this inflection point, the data were divided into two groups. Segmented regression analysis was performed on each group separately and the results were shown in **Supplementary Table 5**.

### Sensitivity analyses

**Figure 3:**
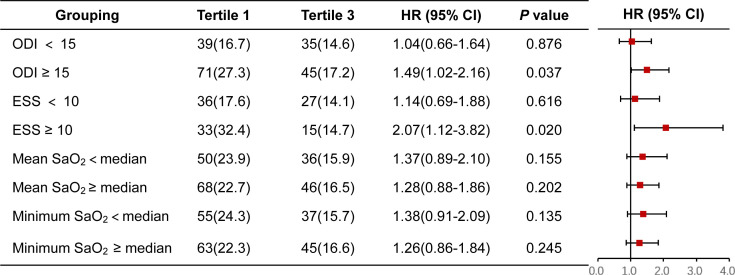
Forest plot for MACCE in FT3/FT4 tertile1 versus FT3/FT4 tertile3 stratified by other OSA-related characteristics. CI, confidence interval; ESS, Epworth sleeping scale; HR, hazard ratio; MACCE, major adverse cardiovascular and cerebrovascular event; ODI, oxygen desaturation index; SaO2, arterial oxygen saturation.

A similar finding was further observed between the lowest and highest tertiles of the FT3/FT4 ratio in patients with ODI ≥ 15 (HR 1.49, 95% CI 1.02-2.16, P = 0.037) and ESS ≥ 10 ((HR 2.07, 95% CI 1.12-3.82, P = 0.020), but not in patients with ODI < 15 or ESS < 10. Regardless of nocturnal hypoxemia indicators (e.g., minimum SaO_2_ and mean SaO_2_), there was no significant difference in the risk of MACCE between the lowest and the highest tertiles of FT3/FT4 ratio (**Figure 3**).

## Discussions

In this study, a significant association was observed between the reduction of FT3/FT4 ratio and an increased risk of MACCE in ACS patients with OSA. This association remained independent after adjustment for confounding risk factors. The incremental risk associated with a lower FT3/FT4 ratio in OSA might be explained by recurrent MI. To our knowledge, the present study is the first one to investigate the prognostic significance of the FT3/FT4 ratio in ACS patients with OSA.

OSA is an independent risk factor for the long-term prognosis of ACS patients (20). In this study, we found that the presence of OSA was associated with a higher incidence of MACCE compared to individuals without OSA among ACS patients. OSA may affect cardiovascular risk through various pathophysiological mechanisms, including fragmented sleep, intrathoracic negative pressure, hypercapnia, and recurrent intermittent hypoxemia. These mechanisms can lead to oxidative stress and systemic inflammation, which can increase the risk of cardiovascular disease (2, 21). However, the precise mechanism through which OSA influences cardiovascular events remains unclear.

TH are important hormones, and due to the widespread presence of TH receptors in myocardial tissue, even subtle variations in the reference range of TH levels can lead to an imbalance in the cardiovascular system. The effects of abnormal TH levels on the cardiovascular system are therefore well recognized (22, 23). The FT3/FT4 ratio reflects the dynamic conversion process of the endocrine system to convert T4 to T3 to gain biological activity and is considered a surrogate indicator for assessing the level of peripheral thyroid hormone deiodination and deiodinase activity (9). Okoye et al. reported that the FT3/FT4 ratio has been proved to be a marker of frailty syndrome and clinical complexity, which is negatively associated with frailty and risk of death in the elderly (24). Brozaitiene et al. found that thyroid hormones (i.e., FT4 level and FT3/FT4 ratio) together with NT-pro-BNP level may be valuable and simple predictors of long-term outcomes of CAD patients after experiencing ACS (25). Wang et al. found that a reduced level of FT3/FT4 ratio was a potential marker of poor prognosis in euthyroid patients with ACS and diabetes after PCI, patients with lower FT3/FT4 levels were older, more female, had a higher prevalence of previous hypertension and lower BMI (26). Our study is consistent with these reports that there was a significant association between a lower FT3/FT4 ratio and a variety of health markers and disease states, with lower BMI, diastolic blood pressure, eGFR, and LVEF, but higher Hs-CRP and prevalence of previous stroke and was more likely to be diagnosed with STEMI at the time of admission. We also found that patients with lower FT3/FT4 levels had a significantly increased risk of cardiovascular death and hospitalization for HF compared with those with higher FT3/FT4 levels. Therefore, the FT3/FT4 ratio may serve as a useful biomarker to help clinicians assess a patient's prognosis for cardiovascular events and may also identify high-risk patients who are in poorer health and have more complex clinical comorbidities, leading to more aggressive preventive measures.

Previous studies have shown that OSA may affect thyroid function and hormone production, and that OSA often coexists with hypothyroidism (27–29). However, several other studies have shown no significant correlation between the severity of OSA and TH levels, including TSH, FT3, and FT4 (30, 31). Therefore, the importance of screening thyroid hormone levels in patients with OSA is controversial. In the present study, we found no significant differences between THs and FT3/FT4 ratios with or without OSA, and furthermore, there were no significant differences in the incidence of OSA and nocturnal sleep monitoring indices regardless of the FT3/FT4 ratio.

In the present study, we observed that a lower FT3/FT4 ratio significantly increased the risk of MACCE in ACS patients with OSA, whereas this association was absent in ACS patients without OSA. This finding may be due to the fact that the OSA population has different degrees of frailty and complexity, such as a higher prevalence of previous stroke, elevated Hs-CRP and reduced eGFR, whereas the non-OSA group is more homogeneous. These differences emphasize the unique, high-risk pathophysiological state of OSA patients. The prognostic value of the FT3/FT4 ratio has been demonstrated in a variety of cardiovascular diseases, including ACS patients with comorbid diabetes mellitus (26) and patients with NSTE-ACS after PCI (32). Low FT3/FT4 ratios have also been shown to significantly increase the long-term risk of cardiovascular death and MACCE in patients with three-branch CAD (14) and non-obstructive coronary artery myocardial infarction (33). Our secondary analyses of MACCE showed no interaction between the presence of OSA and the FT3/FT4 ratio, suggesting that OSA and the FT3/FT4 ratio may be independent factors in the risk of MACCE. In conclusion, these findings suggest that the FT3/FT4 ratio may not only serve as a prognostic indicator for patients with ACS combined with OSA but may also have similar predictive value in ACS populations with other high-risk factors and may facilitate risk stratification in this specific population. However, the generalizability of this finding needs to be further validated in a larger and more diverse patient population.

This study has several limitations. First, this was a single-center cohort study, and potential confounders might not be fully adjusted. Second, the diagnosis of OSA was made by portable polygraphy, which may underestimate the AHI by overestimating actual sleep time. Third, the blood samples were taken at baseline, and thyroid hormone levels undergo change over time, so it is likely that baseline levels do not correspond to follow-up levels. Fourth, we did not mention or analyze the medication usage of the included patients, this is crucial as iodinated contrast agents (including amiodarone and glucocorticoids) may affect thyroid function. Finally, treatment of OSA, especially Continuous Positive Airway Pressure therapy, was not analyzed and followed up, which may have an impact on prognosis.

## Conclusion

In patients with ACS and OSA, lower FT3/FT4 ratios are strongly associated with a significant increase in MACCE. Further validation in a larger, more diverse population is needed to determine the generalizability of this finding.

## Data Availability

The data analyzed in this study is subject to the following licenses/restrictions: Datasets used and/or analyzed in this study are available on reasonable request from the author. Requests to access these datasets should be directed to SN, spnie@ccmu.edu.cn.
