# Supplementary material for: The long-term prognostic implications of free triiodothyronine to free thyroxine ratio in patients with obstructive sleep apnea and acute coronary syndrome
Source: Front Endocrinol (Lausanne). 2024 Sep 16;15:1451645. doi: 10.3389/fendo.2024.1451645 (PMC11439673; doi:10.3389/fendo.2024.1451645)
Supplement: Supplementary file 1 [file DataSheet1.docx]

Supplementary Material

# Supplementary Tables

**Supplementary Table 1. Relationship between OSA and thyroid hormones**

|  | OSA (n=812) | Non-OSA (n=735) | P value |
| --- | --- | --- | --- |
| TSH, mIU/L | 1.67(1.13-2.44) | 1.62(1.11-2.34) | 0.420 |
| FT3, pmol/L | 4.84(4.39-5.26) | 4.81(4.43-5.19) | 0.600 |
| FT4, pmol/L | 11.13(10.09-12.37) | 11.03(10.01-12.19) | 0.184 |
| FT3/FT4 ratio | 0.44(0.38-0.49) | 0.43(0.38-0.49) | 0.501 |

The data is presented as median (first quartile to third quartile). Abbreviations: FT3, free triiodothyronine; FT4, free thyroxine; TSH, thyroid stimulating hormone.

**Supplementary Table 2. Demographic and clinical characteristics according to FT3/FT4 ratio tertiles**

| **Variables** | **Tertile 1 (n=510)** | **Tertile 2 (n=525)** | **Tertile 3 (n=512)** | ***P* value** |
| --- | --- | --- | --- | --- |
| **Subjects** |  |  |  |  |
| Age, years | 59.1±10.3 | 55.1±10.4 | 53.9±10.0 | ＜0.001 |
| Male | 417(81.8) | 456(86.9) | 461(90.0) | 0.001 |
| BMI, kg/m^2^ | 26.2±3.5 | 27.2±3.6 | 27.7±3.6 | ＜0.001 |
| Waist-to-hip ratio | 0.98(0.94-1.01) | 0.98(0.95-1.02) | 0.99(0.95-1.02) | 0.044 |
| Neck circumference, cm | 40(38-42) | 41(39-43) | 41(39-44) | ＜0.001 |
| Systolic BP, mmHg | 125(116-137) | 127(118-140) | 129(120-139) | 0.064 |
| Diastolic BP, mmHg | 74(70-82) | 76(70-84) | 80(70-87) | ＜0.001 |
| **Medical history** |  |  |  |  |
| Diabetes | 248(48.6) | 229(43.6) | 219(42.8) | 0.126 |
| Hypertension | 363(71.2) | 372(70.9) | 351(68.6) | 0.606 |
| Hyperlipidemia | 157(30.8) | 187(35.6) | 181(35.4) | 0.184 |
| Prior stroke | 77(15.1) | 54(10.3) | 41(8.0) | 0.001 |
| Prior MI | 84(16.5) | 94(17.9) | 78(15.2) | 0.511 |
| Prior PCI | 114(22.4) | 106(20.2) | 101(19.7) | 0.543 |
| Prior CABG | 10(2.0) | 5(1) | 6(1.2) | 0.339 |
| Current Smoking | 220(43.1) | 269(51.2) | 265(51.8) | 0.005 |
| **Baseline tests** |  |  |  |  |
| eGFR, mL·min-1·1.73 m^-2^ | 102.4(84.4-119.2) | 105.3(90.6-122.6) | 107.9(92.1-123.5) | 0.001 |
| Hemoglobin A1C, mmol/L | 6.1(5.6-7.3) | 6(5.6-6.9) | 6.1(5.6-6.9) | 0.088 |
| Hs-CRP, mg/L | 2.36(0.81-8.17) | 2.07(0.81-5.37) | 1.77(0.70-4.54) | 0.003 |
| BNP, pg/mL | 65.9(26.0-183.0) | 49.1(19.9-120.5) | 39(19.9-91.74) | ＜0.001 |
| LVEF, % | 60(55-65) | 61(58-65) | 63(58-66) | 0.001 |
| Total cholesterol, mmol/L | 3.99(3.41-4.80) | 4.15(3.49-4.95) | 4.26(3.57-4.99) | 0.002 |
| Triglyceride, mmol/L | 1.36(1.01-1.92) | 1.61(1.16-2.3) | 1.65(1.23-2.53) | ＜0.001 |
| LDL-C, mmol/L | 2.36(1.79-3.02) | 2.44(1.91-3.13) | 2.49(1.95-3.11) | 0.038 |
| HDL-C，mmol/L | 0.99(0.85-1.17) | 0.98(0.84-1.15) | 1.01(0.87-1.16) | 0.213 |
| TSH, mIU/L | 1.63(1.10-2.42) | 1.64(1.15-2.38) | 1.67(1.13-2.41) | 0.826 |
| FT3, pmol/L | 4.42(4.10-4.78) | 4.84(4.51-5.16) | 5.18(4.84-5.59) | ＜0.001 |
| FT4, pmol/L | 12.47(11.52-13.56) | 11.14(10.38-11.97) | 9.83(8.99-10.52) | ＜0.001 |
| **Diagnosis** |  |  |  | ＜0.001 |
| STEMI | 150(29.4) | 104(19.8) | 85(16.6) |  |
| NSTEMI | 91(17.8) | 114(21.7) | 83(16.2) |  |
| UA | 269(52.7) | 307(58.5) | 344(67.2) |  |
| **Procedures** |  |  |  |  |
| PCI | 316(62.0) | 329(62.7) | 321(62.7) | 0.963 |
| CABG | 40(7.8) | 37(7) | 35(6.8) | 0.807 |
| **Medications on discharge** |  |  |  |  |
| Aspirin | 491(96.3) | 513(97.7) | 502(98.0) | 0.172 |
| P2Y_12_ inhibitors | 469(92.0) | 478(91) | 467(91.2) | 0.856 |
| β-Blockers | 394(77.3) | 409(77.9) | 388(75.8) | 0.708 |
| ACEIs/ARBs | 323(63.3) | 328(62.5) | 307(60.0) | 0.513 |
| Statins | 504(98.8) | 514(97.9) | 502(98.0) | 0.48 |

The data is presented as mean ± SD, median (first quartile to third quartile), or n (%). Abbreviations: ACEI, angiotensin-converting enzymes inhibitor; ARB, angiotensin receptor blocker; BMI, body mass index; BP, blood pressure; CABG, coronary artery bypass grafting; FT3, free triiodothyronine; FT4, free thyroxine; Hs-CRP, High sensitivity C-reactive protein; HDL-C, high-density lipoprotein cholesterol; LDL-C, low-density lipoprotein cholesterol; LVEF, left ventricular ejection fraction; NSTEMI, Non-ST-segment-elevation myocardial infarction; PCI, percutaneous coronary intervention; STEMI, ST-segment-elevation myocardial infarction; TSH, thyroid stimulating hormone; UA, unstable angina.

**Supplementary Table 3. Overnight sleep monitoring results according to FT3/FT4 ratio tertiles**

| **Variables** | **Tertile 1 (n=510)** | **Tertile 2 (n=525)** | **Tertile 3 (n=512)** | ***P* value** |
| --- | --- | --- | --- | --- |
| **Sleep study** |  |  |  |  |
| AHI ≥ 15 | 268(52.5) | 281(53.5) | 263(51.4) | 0.785 |
| AHI, events·h^−1^ | 16(8-29) | 17(8-30) | 15(8-30) | 0.841 |
| ODI, events·h^−1^ | 15.9(8.6-28.3) | 16(8.5-28.9) | 16.5(8.7-28.7) | 0.831 |
| Minimum SaO_2_, % | 85(80-88) | 86(82-88) | 85(80-88) | 0.136 |
| Mean SaO_2_, % | 94(93-95) | 94(93-95) | 94(93-95) | 0.055 |
| T90, % | 2.1(0.3-10.0) | 2(0.3-9) | 2.6(0.4-11.0) | 0.265 |
| Epworth Sleepiness Scale | 7(4-11) | 7(4-12) | 7(4-11) | 0.679 |

The data is presented as n (%) or median (first quartile to third quartile); AHI, apnea-hypopnea index; ODI, oxygen desaturation index; SaO2, arterial oxygen saturation; T90=percentage of Time with SaO2 ＜90%.

**Supplementary Table 4. Clinical outcomes** **according to FT3/FT4 ratio tertiles**

| **Clinical outcomes** | **Group** | **Events (n, %)** | **HR (95% CI)** | ***P* value** |
| --- | --- | --- | --- | --- |
| **MACCE** | Tertile 1 | 118(23.1) | 1.31(0.99-1.74) | 0.060 |
|  | Tertile 2 | 105(20.0) | 1.20(0.90-1.60) | 0.216 |
|  | Tertile 3 | 83(16.2) | 1 (Ref) |  |
| **Recurrent MI** | Tertile 1 | 17(3.3) | 1.45(0.66-3.18) | 0.353 |
|  | Tertile 2 | 13(2.5) | 1.24(0.54-2.82) | 0.616 |
|  | Tertile 3 | 10(2.0) | 1 (Ref) |  |
| **Cardiovascular death** | Tertile 1 | 15(2.9) | 3.37(1.12-10.17) | 0.031 |
|  | Tertile 2 | 10(1.9) | 2.41(0.76-7.69) | 0.137 |
|  | Tertile 3 | 4(0.8) | 1 (Ref) |  |
| **Hospitalization for UA** | Tertile 1 | 66(12.9) | 1.00(0.70-1.41) | 0.977 |
|  | Tertile 2 | 76(14.5) | 1.17(0.84-1.64) | 0.355 |
|  | Tertile 3 | 61(11.9) | 1 (Ref) |  |
| **Stroke** | Tertile 1 | 16(3.1) | 1.46(0.66-3.23) | 0.347 |
|  | Tertile 2 | 13(2.5) | 1.24(0.55-2.83) | 0.606 |
|  | Tertile 3 | 10(2) | 1 (Ref) |  |
| **Ischemia-driven revascularization** | Tertile 1 | 46(9) | 1.10(0.72-1.70) | 0.655 |
|  | Tertile 2 | 40(7.6) | 0.99(0.63-1.54) | 0.956 |
|  | Tertile 3 | 38(7.4) | 1 (Ref) |  |
| **Hospitalization for HF** | Tertile 1 | 14(2.7) | 10.90(1.43-83.19) | 0.021 |
|  | Tertile 2 | 3(0.6) | 2.86(0.30-27.52) | 0.362 |
|  | Tertile 3 | 1(0.2) | 1 (Ref) |  |
| **All-cause death** | Tertile 1 | 16(3.1) | 1.80(0.77-4.22) | 0.175 |
|  | Tertile 2 | 15(2.9) | 1.81(0.77-4.26) | 0.177 |
|  | Tertile 3 | 8(1.6) | 1 (Ref) |  |

Data are presented as n (%). CI, Confidence Interval; HF, hear failure; HR, hazard ratio; MACCE, major adverse cardiovascular and cerebrovascular event; MI, myocardial infarction; UA, unstable angina.

**Supplementary Table 5. Effect of standardized FT3/FT4 ratio level on survival**

| **Groups** | | **HR per SD** | **95% CI** | ***P* value** |
| --- | --- | --- | --- | --- |
| Overall | FT3/FT4 < 0.5 | 0.80 | 0.69, 0.93 | 0.003 |
|  | FT3/FT4 ≥ 0.5 | 0.79 | 0.25, 2.52 | 0.700 |
| OSA | FT3/FT4 < 0.5 | 0.84 | 0.75, 0.94 | 0.003 |
|  | FT3/FT4 ≥ 0.5 | 0.89 | 0.55, 1.42 | 0.620 |
| Non-OSA | FT3/FT4 < 0.5 | 0.91 | 0.76, 1.10 | 0.330 |
|  | FT3/FT4 ≥ 0.5 | 0.96 | 0.61, 1.50 | 0.850 |

CI, Confidence Interval; HR, hazard ratio.

## Supplementary Figures

##
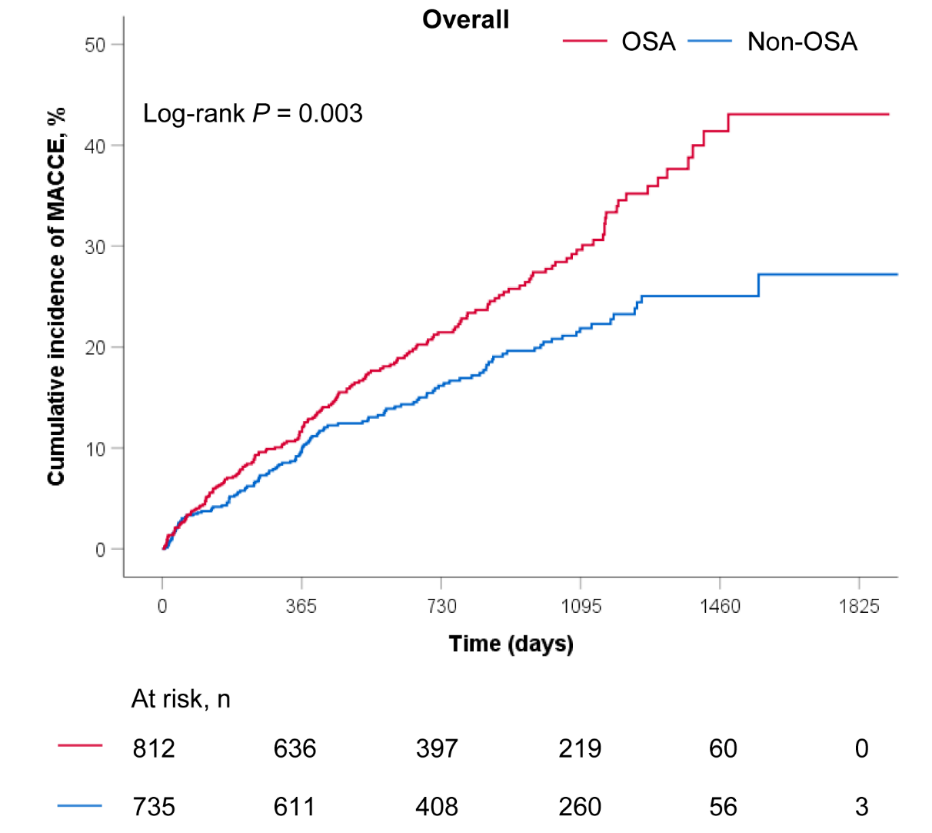


## Supplementary Figure 1. Association of the FT3/FT4 ratio with MACCE by unadjusted restricted cubic splines in (A) overall patients, (B) patients with OSA, and (C) patients without OSA. MACCE, major adverse cardiovascular and cerebrovascular event.


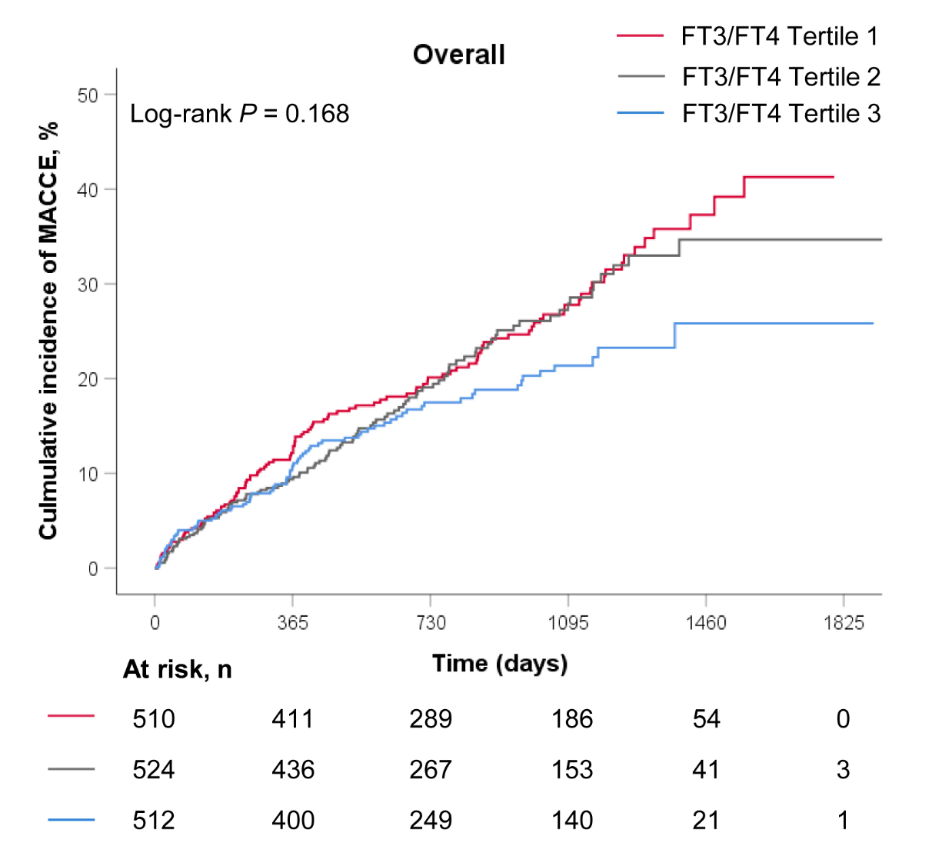


**Supplementary Figure 2**. Kaplan-Meier curves in OSA versus non-OSA groups MACCE. MACCE, major adverse cardiovascular and cerebrovascular event.


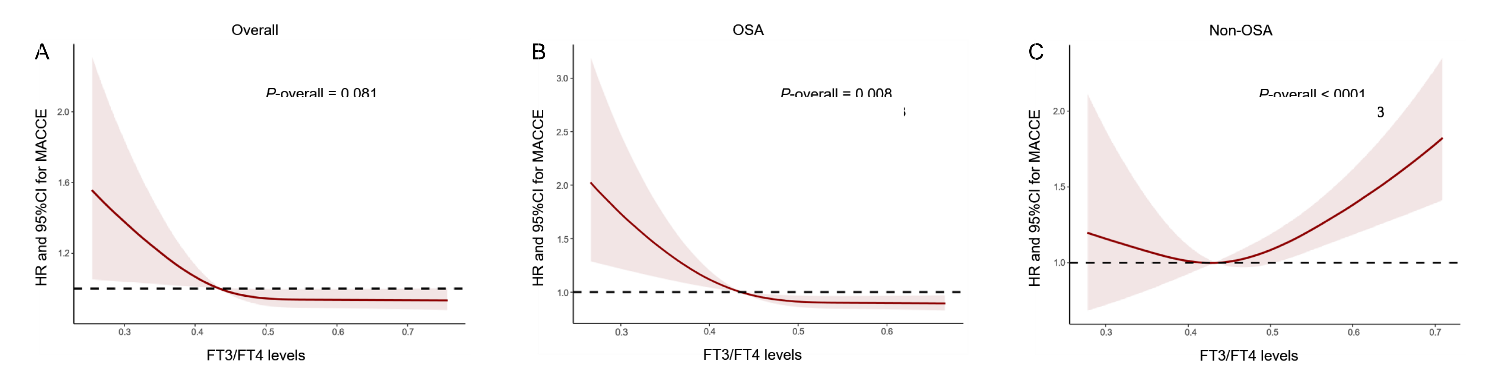


**Supplementary Figure 3**. Kaplan-Meier curves in FT3/FT4 tertiles MACCE. MACCE, major adverse cardiovascular and cerebrovascular event.
