# Supplementary figures and images for: The long-term prognostic implications of free triiodothyronine to free thyroxine ratio in patients with obstructive sleep apnea and acute coronary syndrome
Source: Front Endocrinol (Lausanne). 2024 Sep 16;15:1451645. doi: 10.3389/fendo.2024.1451645 (PMC11439673; doi:10.3389/fendo.2024.1451645)

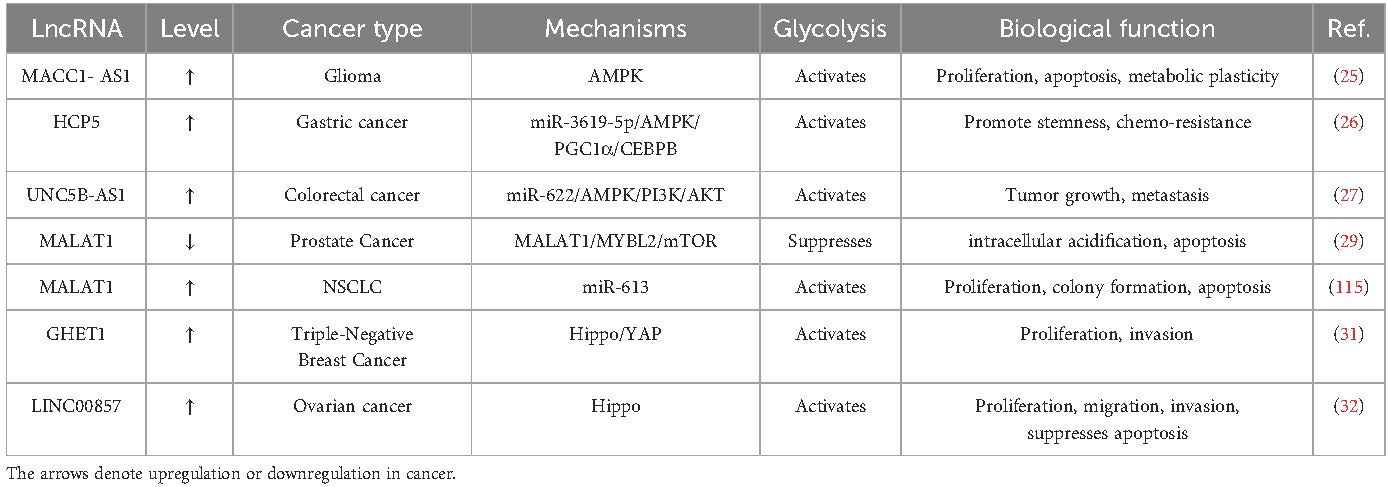

Supplement: Supplementary file 2 [file Table1.jpg]

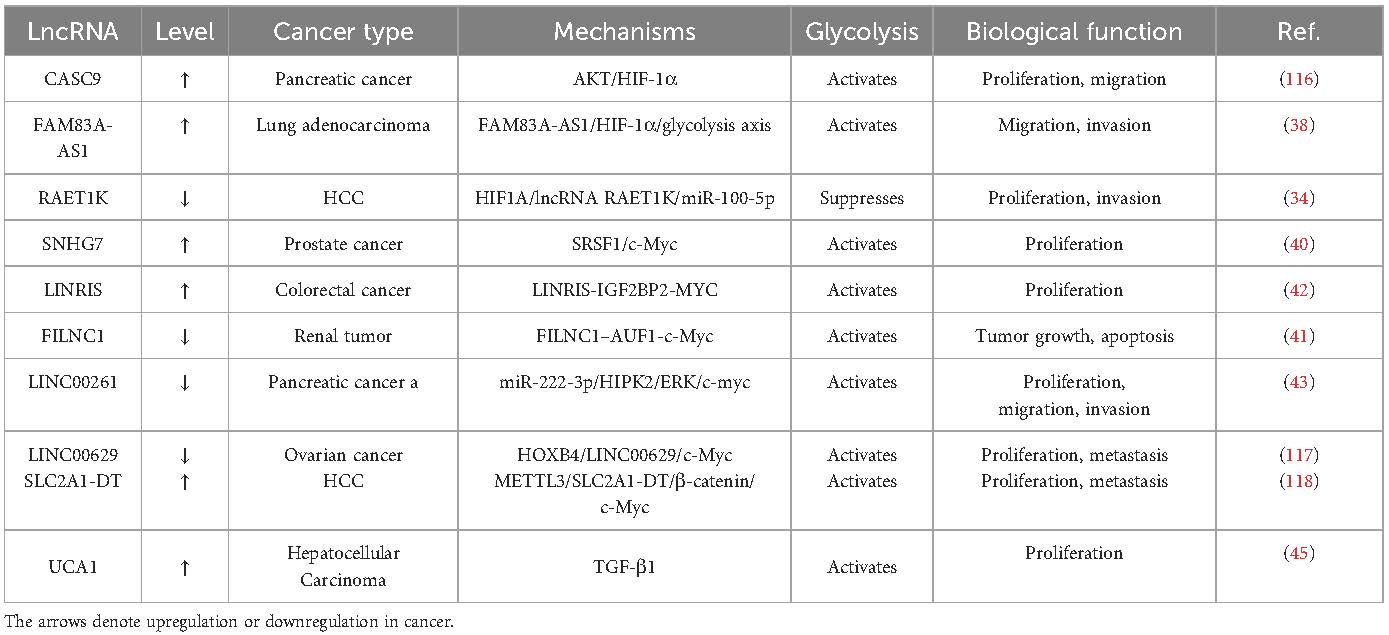

Supplement: Supplementary file 3 [file Table2.jpg]

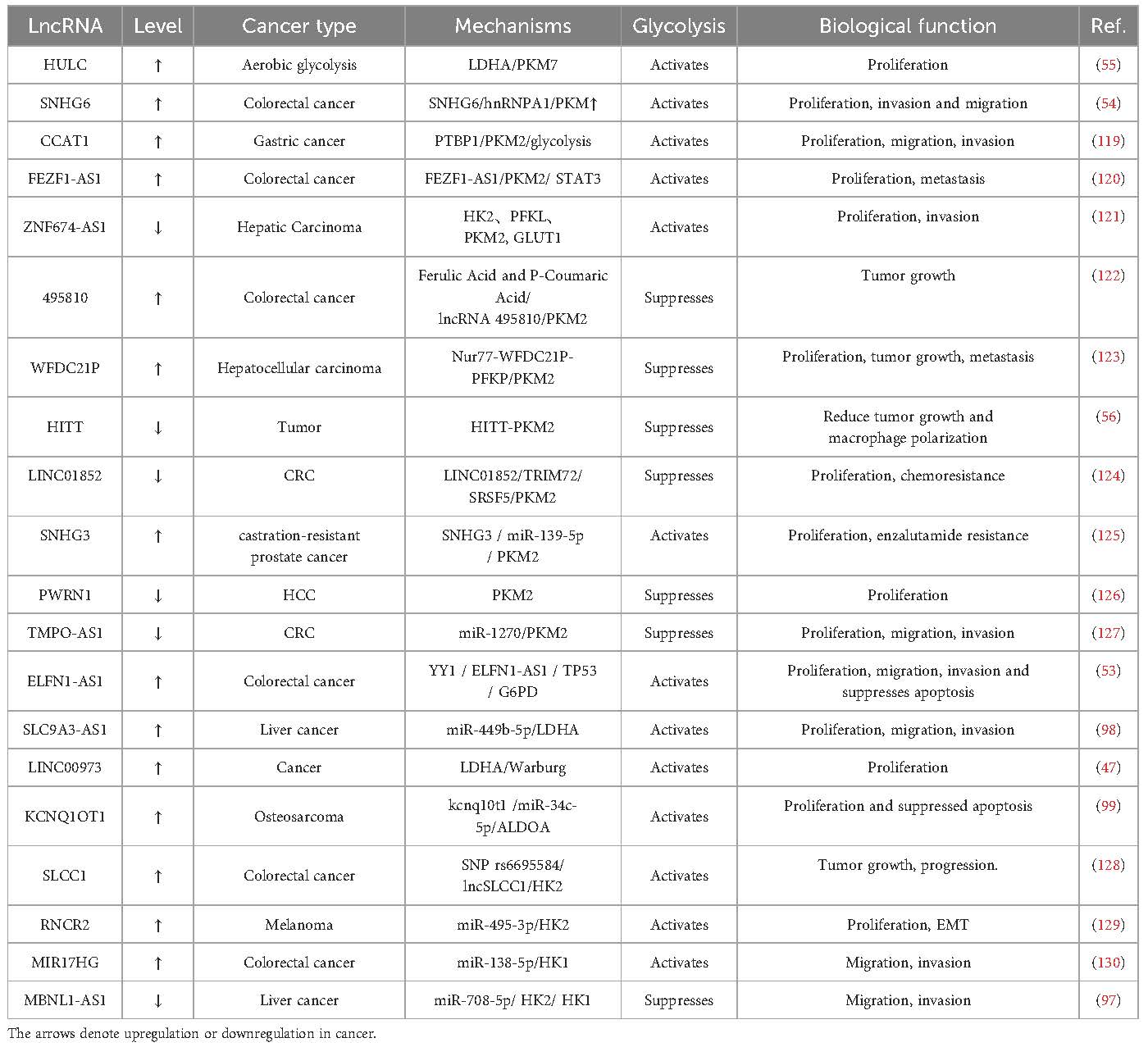

Supplement: Supplementary file 4 [file Table3.jpg]

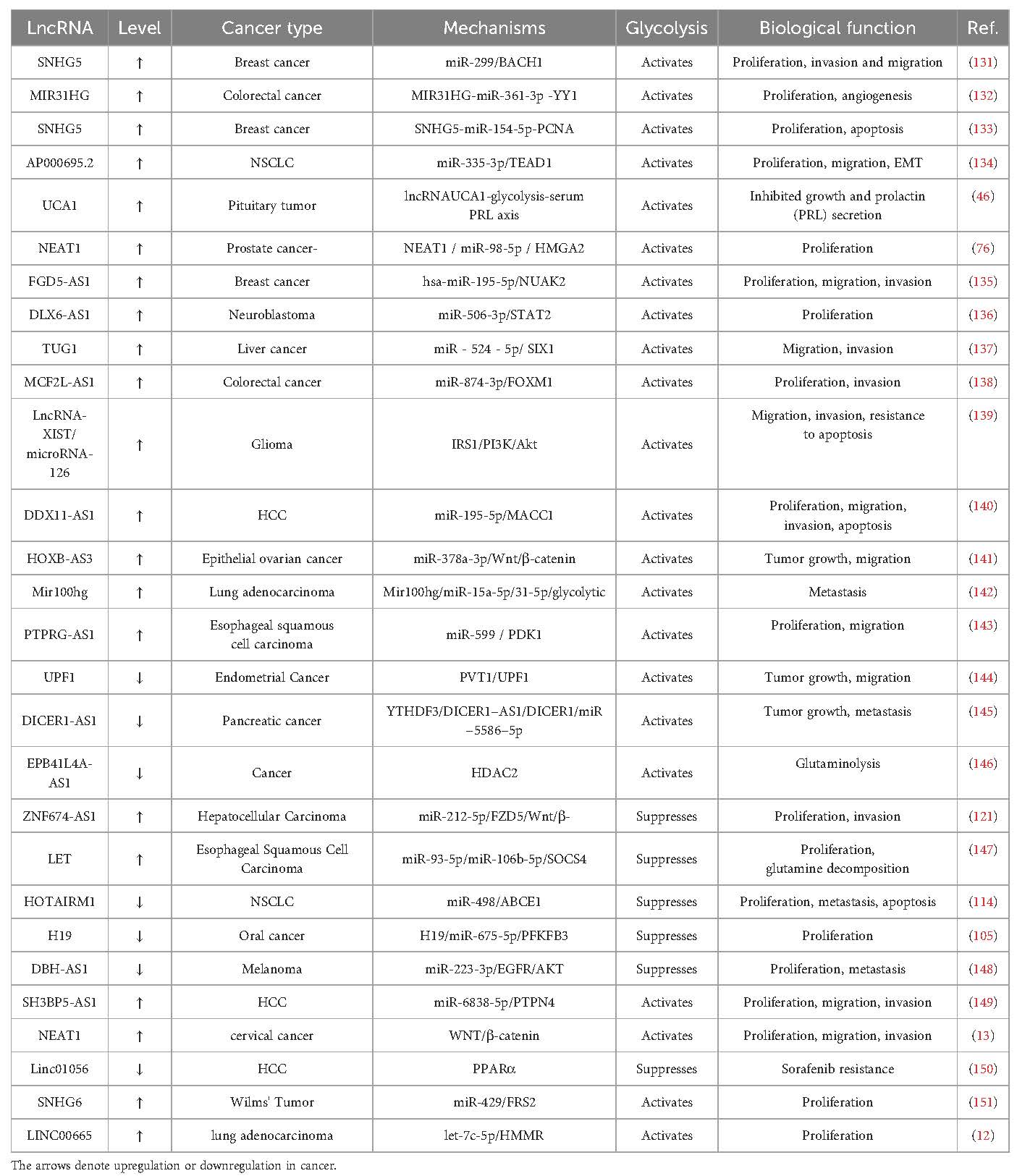

Supplement: Supplementary file 5 [file Table4.jpg]
